# The complications of cyclosporine a in pediatric use and its effectiveness in treating pediatric congenital heart diseases-a meta analysis in combined with a retrospective clinical study

**DOI:** 10.3389/fphar.2025.1727970

**Published:** 2025-11-27

**Authors:** Yiting Xue, Siqi She, Sijuan Sun, Junxian Chen, Lincai Ye, Yanhui Huang

**Affiliations:** 1 Department of Thoracic and Cardiovascular Surgery, Shanghai Children’s Medical Center, Shanghai Jiao Tong University School of Medicine, Shanghai, China; 2 Department of Pediatric Intensive Care Unit, Shanghai Children’s Medical Center, Shanghai Jiao Tong University School of Medicine, Shanghai, China; 3 Thoracic and Cardiovascular Surgery, The Affiliated Women and Children’s Hospital of Ningbo University, Ningbo, Zhejiang, China; 4 Institute of Pediatric Translational Medicine, Shanghai Children’s Medical Center, Shanghai Jiao Tong University School of Medicine, Shanghai, China; 5 Shanghai Institute for Pediatric Congenital Heart Disease, Shanghai Children’s Medical Center, Shanghai Jiao Tong University School of Medicine, Shanghai, China; 6 Department of Anesthesiology, Shanghai Children’s Medical Center, Shanghai Jiao Tong University School of Medicine, Shanghai, China

**Keywords:** reduced pulmonary blood perfusion, volume overload, children, cyclosporine A (CsA), congenital heart diseases (CHDs)

## Abstract

**Background:**

Advances in surgical techniques have significantly enhanced perioperative survival rates in children with congenital heart diseases (CHDs). However, a growing population of adolescent and adult CHD survivors now face long-term challenges, including increased susceptibility to arrhythmia and impaired exercise tolerance. Preclinical studies suggested that these issues may stem from deficits in lung and cardiomyocyte maturation, driven by neonatal immune activation. The immunosuppressant cyclosporine A (CsA) has shown potential in correcting these maturational deficits in animal models. Nevertheless, the pediatric safety profile of CsA and its therapeutic efficacy for CHDs in clinical setting remain poorly characterized.

**Methods:**

We first conducted a meta-analysis to evaluate complication rates associated with CsA treatment in pediatric populations. We then performed a large, single center retrospective study of 2,439 children treated with CsA to evaluate: (1) the incidence of complications, (2)relevant laboratory parameters, and (3) its therapeutic efficacy in CHD cases.

**Results:**

Our meta-analysis identified infection (55.76%), hirsutism (28.34%), and upper respiratory tract infection (20.22%) as the three most frequent complications observed in CsA-treated pediatric patients. In our clinical cohort (98.97% Han ethnicity; 72.24% from East China), aplastic anemia was the primary indication for CsA treatment (44.73%). Consistent with the meta-analysis, infection remained the predominant complication (overall incidence:17.02%; accounting for 55.78% of all complicated cases). The most commonly detected pathogens were cytomegalovirus (CMV, 7.95%), Epstein-Barr virus (EBV, 5.78%), and herpesvirus (3.6%). Furthermore, following CsA administration, we observed significant elevations in two key infection biomarkers C-reactive protein (CRP) and procalcitonin (PCT). Among these 2,439 patients, CsA was primarily used for immunosuppression in bone marrow transplantation for leukemia, and autoimmune diseases. Sixteen of these patients had CHDs. In this CHD subgroup, CsA treatment was associated with significant improvements in left ventricular ejection fraction (LVEF) and fractional shortening (LVFS) (both p < 0.05).

**Conclusion:**

To our knowledge, this is the largest pediatric study on CsA to date. It demonstrates that immunosuppressants like CsA are rarely used specifically to treat children with isolated CHDs. During CsA therapy in children, infection may emerge as the most critical complication requiring vigilant monitoring. Notably, our findings provide preliminary clinical evidence supporting the potential of immunosuppressive therapy to ameliorate CHD-associated cardiomyocyte maturation deficits.

**Clinical Trial Registration:**

https://www.chictr.org.cn/, identifier ChiCTR2500103672.

## 1 Clinical perspective

What’s new: This study provides the first preliminary evidence characterizing the efficacy of cyclosporine A (CsA) in pediatric congenital heart diseases (CHDs) patients.

What are the clinical implications: Early immunosuppressant treatment may offer a strategy to prevent CHD-associated cardiomyocyte maturation deficits.

## 2 Introduction

Congenital heart diseases (CHDs) represent the most prevalent birth defects worldwide. While advancements in surgical techniques and intensive care have substantially improved perioperative survival rates in children with CHDs (Van Bulck et al., 2022; Crea, 2023), a growing population of adolescent and adult CHD patients now faces significant challenges, including increased arrhythmia susceptibility and impaired exercise endurance (Cai et al., 2023; O'Leary et al., 2024; Lai and Braverman, 2022; Scheffers et al., 2021). These complications profoundly impact patients’ quality of life, making the elucidation of underlying pathophysiological mechanisms and the development of effective therapeutic interventions critical priorities in CHD management.

The postnatal development of cardiac and pulmonary systems remains incomplete in children (Zhou et al., 2024; Zheng and Ye, 2024; Dong et al., 2023; Li et al., 2024), with abnormal hemodynamics - a hallmark of CHDs (Zheng and Ye, 2024) - significantly influencing this maturation process. Among various hemodynamic abnormalities, reduced pulmonary blood perfusion (RPF) and volume overload (VO) emerge as particularly consequential (Li et al., 2024; Li et al., 2023; Brida et al., 2022). Clinical observations reveal that RPF predominantly compromises exercise capacity (Li et al., 2023; Fabi et al., 2020), while VO substantially elevates arrhythmia risk in adulthood compared to healthy populations (Dong et al., 2023; Fabi et al., 2020; Egbe et al., 2019; Vô et al., 2023). Preclinical investigations using neonatal mouse models have demonstrated that both RPF and VO trigger immune-mediated disruptions in organ maturation: RPF impairs alveolar development, while VO adversely affects cardiomyocyte maturation (Zhou et al., 2024; Li et al., 2024; Li et al., 2023; Hu et al., 2022; Sun et al., 2021; Cui et al., 2021). These findings prompted the experimental use of the immunosuppressant cyclosporine A (CsA) to mitigate RPF-associated alveolar dysplasia and VO-induced cardiomyocyte immaturity (Li et al., 2023; Hu et al., 2022; Sun et al., 2021). And cardiomyocyte immaturity has been shown to increase the incidence of arrhythmia (vFeyen et al., 2020; Marchiano et al., 2023).

Despite CsA’s established role in pediatric nephrology, dermatology, and transplant medicine for immune suppression (Robert et al., 2010; Chu et al., 2023; Li et al., 2019), comprehensive safety data specific to pediatric applications remain scarce. The current study addresses this knowledge gap through a dual approach: (1) a systematic meta-analysis evaluating CsA-associated adverse effects in pediatric populations, followed by (2) a 15-year retrospective analysis (2009–2023) of CsA-treated patients at a major pediatric center, with particular focus on complication profiles and therapeutic efficacy in CHD management.

We hypothesized that CsA may improve cardiac function in pediatric CHD patients by modulating immune-mediated cardiomyocyte maturation, and that its safety profile in children is characterized by a high burden of infectious complications requiring proactive management.

## 3 Materials and methods

### 3.1 Ethical statement

All study procedures complied with the ethical principles of the Declaration of Helsinki and received approval from the Animal Welfare and Human Studies Committee at Shanghai Children’s Medical Center (SCMC) (IRB No.: SCMCIRB-K2024265-1). Patients or the public WERE NOT involved in the design, or conduct, or reporting, or dissemination plans of our research.

### 3.2 Study design

**FIGURE 1:**
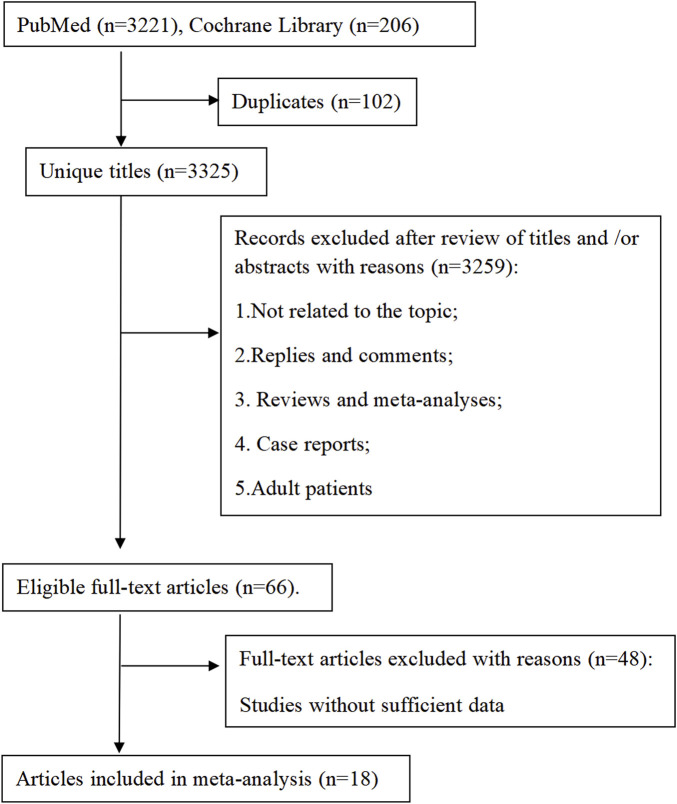
Meta analysis flowchart.

**FIGURE 2:**
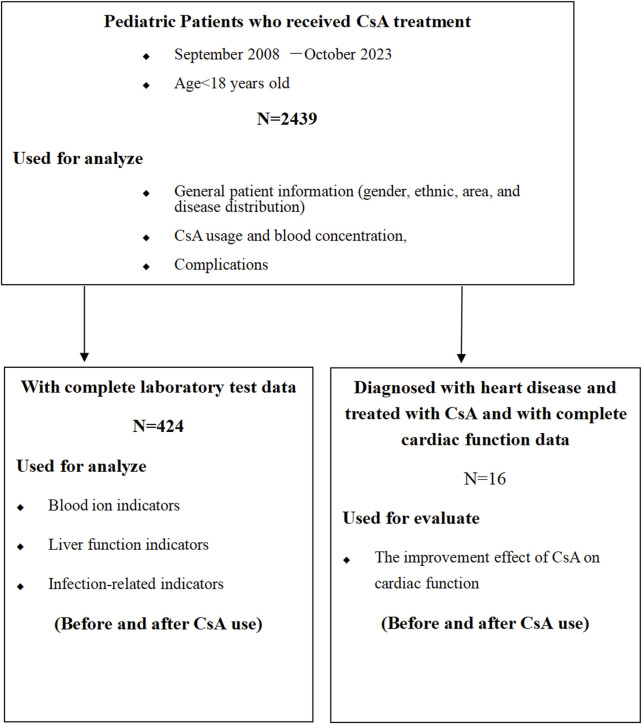
Clinical retrospective analysis flowchart.

The investigation employed a two-phase approach: 1) A systematic meta-analysis evaluating CsA-associated adverse effects in pediatric populations (Figure 1); 2) A retrospective cohort study assessing CsA safety and efficacy in pediatric CHD treatment (Figure 2)

### 3.3 Part I meta-analysis

#### 3.3.1 Data sources and search strategy

We systematically searched PubMed and Cochrane Library for English-language publications through 15 July 2024, using the following search algorithm:

(“Cyclosporin A” OR “Cyclosporin” OR “cyclosporine a OR “cyclosporine” OR “ciclosporin”) AND (“Side effects” OR “adverse effects” OR “complications”). Filters: Age 0–18 years.

#### 3.3.2 Inclusion criteria

Studies were eligible if they: (1) Enrolled pediatric populations (0–18 years); (2) Had ≥10 participants per study arm; (3) Provided explicit complication data; (4) Represented original research articles.

#### 3.3.3 Exclusion criteria

We excluded studies with: (1) Insufficient data for statistical analysis; (2) Non-research publications (reviews, editorials, letters); (3) Duplicate datasets (retaining only the most comprehensive report); (4) Methodological inconsistencies or logical flaws.

#### 3.3.4 Data extraction and quality assessment

Two independent investigators performed literature screening and data extraction using standardized forms. Extracted parameters included:first author, publication year, study characteristics (region, design), population demographics (age, sample size), clinical outcomes. Discrepancies were resolved through consensus discussion. We assessed study quality using Cochrane risk-of-bias tools (Armijo-Olivo et al., 2012).

#### 3.3.5 Statistical analysis

Primary outcome was complication incidence, analyzed using Freeman-Tukey double arcsine transformation (Chen et al., 2023). We evaluated heterogeneity using:Cochran’s Q statistic (significance threshold p < 0.05) and I^2^ index (>50% indicating substantial heterogeneity). For heterogeneous data (I^2^ >50% or Q p < 0.05), we applied random-effects models; otherwise, fixed-effects models were used (Fernández-Pérez et al., 2023). Publication bias was assessed via Egger’s and Begg’s tests (significance threshold p < 0.05 for both) (significance threshold p < 0.05 for both) (Kvaskoff et al., 2021). All analyses were performed using R 4.2.1 software (R Foundation for Statistical Computing, Beijing, China, Package: Meta).

### 3.4 Part II retrospective cohort study

#### 3.4.1 Patients and study design

We analyzed 2,439 consecutive pediatric patients (<18 years) who received CsA therapy at Shanghai Children’s Medical Center (SCMC) (September 2009-October 2023). Our institutional protocol for CsA therapy is guided by therapeutic drug monitoring to maintain a target trough level of 100–200 ng/mL, thus balancing efficacy and toxicity. Adjunctive infection-control measures, including antiviral/antifungal prophylaxis for high-risk individuals and ongoing clinical surveillance, are routinely implemented to minimize opportunistic infections.

The study comprised three analytical tiers:

1. Demographic analysis (n = 2,439): gender distribution; ethnic composition; diagnostic profiles; geographic origins; complication documentation.
2. Laboratory parameter analysis (n = 424, with complete pre-vs. post-CsA administration data): electrolyte profiles; hepatic function markers; infection biomarkers.
3. Cardiac function assessment and CsA pharmacokinetics (dosage and blood levels) (n = 16 CHD patients, only 16 patients developed CHDs and were treated with CsA). Echocardiographic assessments were performed using a Philips i.e., 33 systems (Philips, Bothell, WA) by experienced pediatric cardiologists following a standardized protocol, with measurements averaged over three consecutive cardiac cycles.

#### 3.4.2 Statistical analysis

Continuous variables following a normal distribution were expressed as mean ± standard deviation (SD), and analyzed with paired Student’s t-test. Categorical variables and non-normally distributed continuous variables were presented as the median and interquartile range [M (P25, P75)] and analyzed with Wilcoxon matched-pairs signed rank test. All statistical analyses were conducted using GraphPadPrism version 9.3.1.

## 4 Results

### 4.1 Part I meta-analysis

#### 4.1.1 Study characteristics

Our systematic search identified 3,427 publications, of which 18 articles (comprising 23 studies) met inclusion criteria (Figure 1). The analyzed studies (Supplementary Table S1) investigated CsA in: nephrotic syndrome (8 studies, 34.8%), kidney transplantation (7 studies, 30.4%), aplastic anemia (3 studies, 13.0%), atopic dermatitis (3 studies, 13.0%), and other indications (2 studies, 8.7%). Notably, no studies reported CsA use for cardiopulmonary diseases. The publications (1998–2023) originated from seven countries and collectively enrolled 1,013 pediatric patients (range: 12–100 per study).

#### 4.1.2 Complication profile

Our analysis identified 16 distinct complications during CsA therapy in pediatric patients (Supplementary Table S2), which we categorized as: 1) Most prevalent complications: infection: 55.76% (95% CI: 31.17–79.00), hirsutism: 28.34% (95% CI: 18.38–39.40), and upper respiratory tract infection: 20.22% (95% CI: 12.24–29.45); 2) Other notable adverse events: Gastrointestinal (abdominal pain, diarrhea, nausea, and vomiting), hematologic (anemia, leukopenia, and neutropenia), neurologic (headache, tremor, and psychiatric disorders), and other (gingival hyperplasia, hypertension, ileus, and joint pain). The particularly high infection rate (55.76%) during CsA treatment suggested that there may be a need for rigorous infection prophylaxis, especially given the inherent immunological vulnerability of pediatric patients [45].

#### 4.1.3 Heterogeneity and bias assessment

**FIGURE 3:**
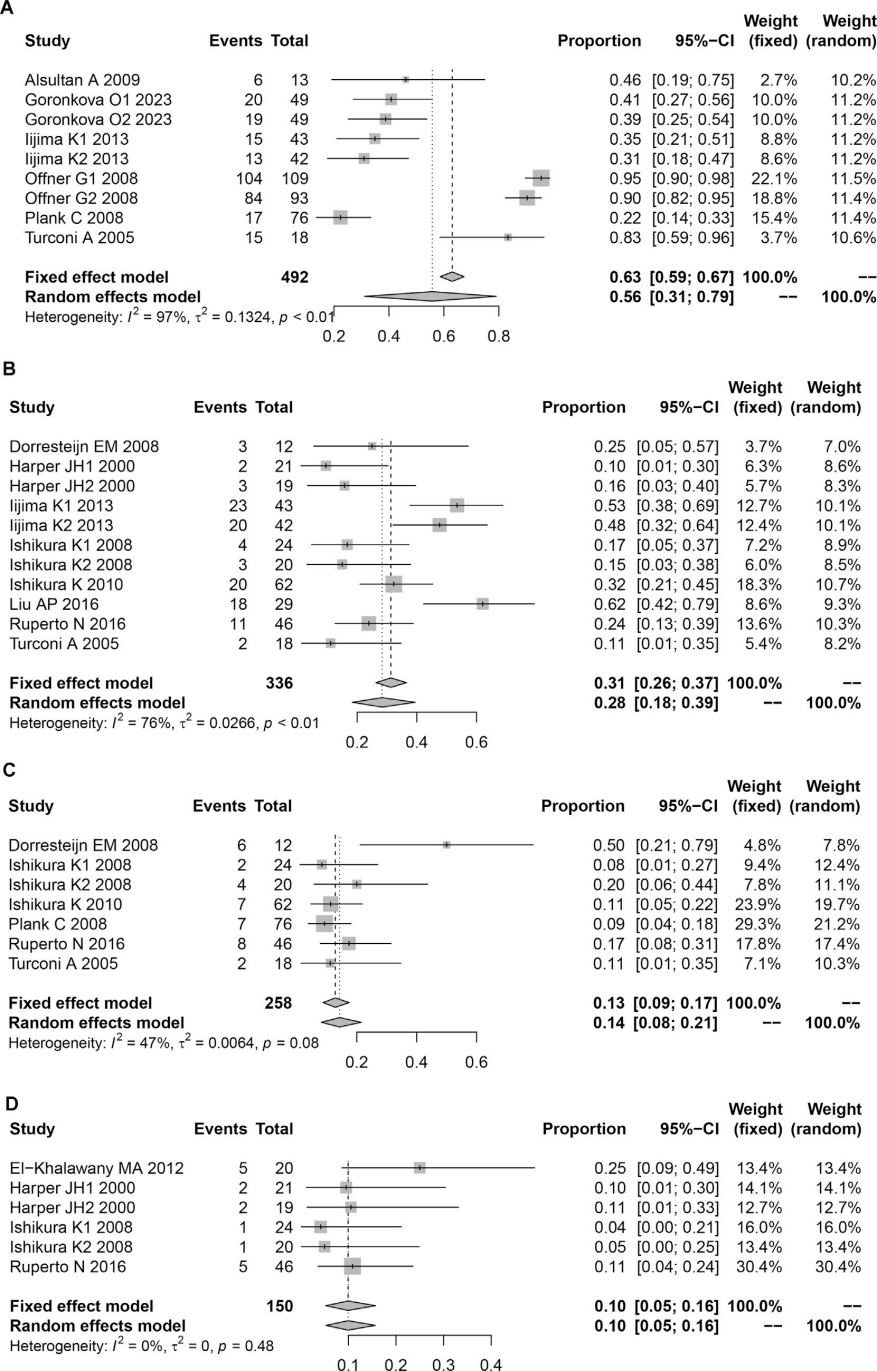
Forest plot of complications in children associated with CsA treatment. **(A)** Infection. **(B)** Hirsutism. **(C)** Gingival hyperplasia. **(D)** Headac.

Our heterogeneity analysis revealed distinct patterns across complication types (Supplementary Table S2; Figure 3; Supplementary Figures S1–S3). Four outcomes demonstrated low heterogeneity (I^2^ <50%): gingival hyperplasia (I^2^ = 42%), headache (I^2^ = 38%), ileus (I^2^ = 29%), and tremor (I^2^ = 17%), warranting fixed-effects model analysis. All remaining variables showed substantial heterogeneity (I^2^ >50%), necessitating random-effects models.

Publication bias assessment (Supplementary Table S3; Supplementary Figures S1–S3) identified significant bias for three outcomes: upper respiratory tract infection, nausea, and neutropenia. No significant publication bias was detected for other complications.

In summary, the meta-analysis revealed CsA’s predominant use in pediatric kidney transplantation and nephrotic syndrome, with no reported applications for cardiopulmonary conditions. Most frequent complications were infection (55.76%), hirsutism (28.34%), and upper respiratory tract infection (20.22%). In addition, there were no severe developmental complications observed, suggesting that overall safety profile supports pediatric use with appropriate monitoring.

### 4.2 Part II single center-retrospective cohort study

#### 4.2.1 Demographic characteristics

**FIGURE 4:**
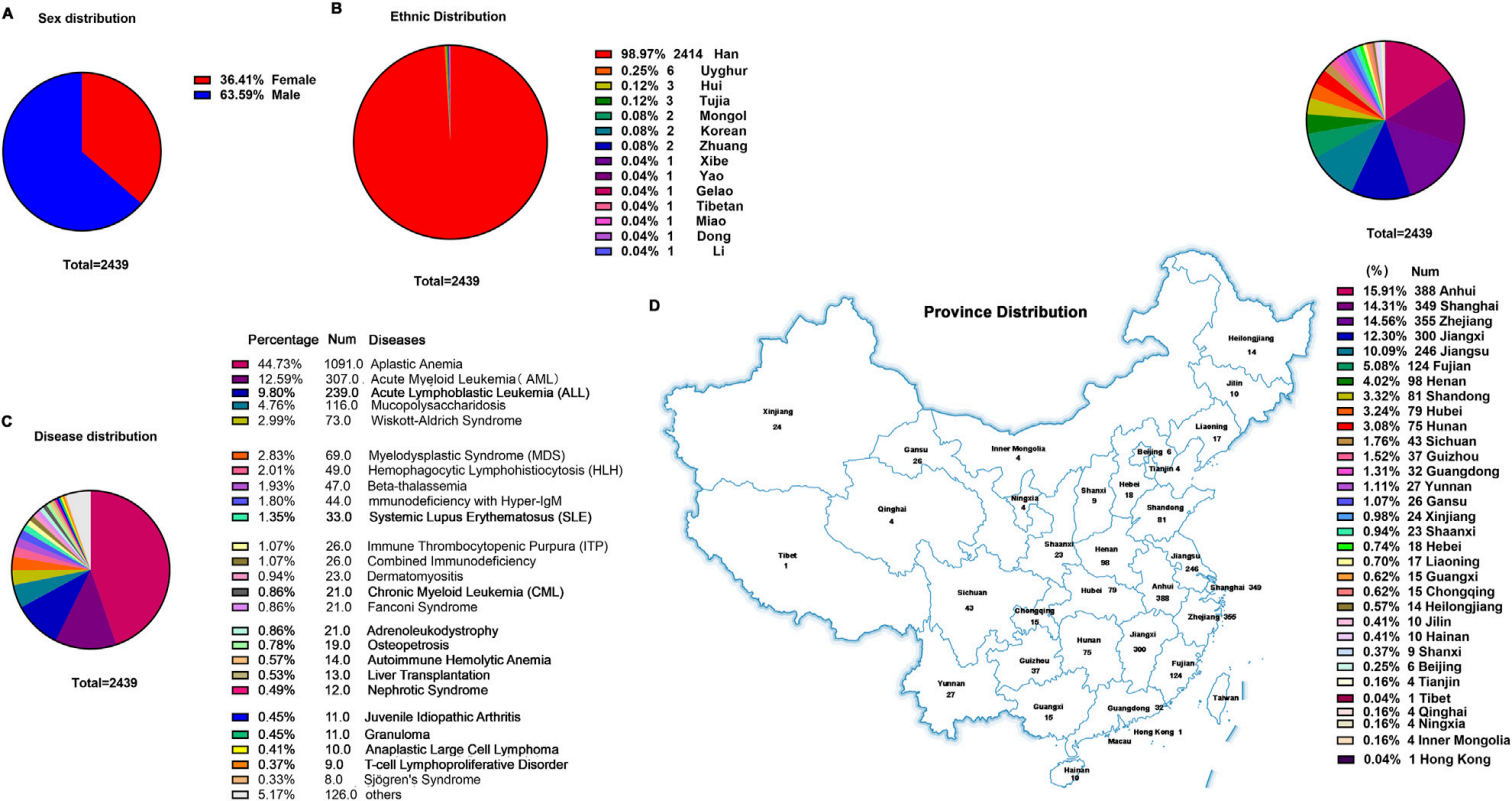
General clinical information of children treated with CsA. **(A)** Sex distribution. **(B)** Ethnic distribution. **(C)** Disease distribution. **(D)** Province distribution.

The cohort comprised 2,439 patients, with a male predominance (63.59% vs. 36.41% female; Figure 4A), a demographic skew potentially attributable to China’s one-child policy implemented between 1980–2015. Ethnic distribution revealed Han Chinese as the majority (98.97%), followed by Uyghur (0.25%), Hui (0.12%), and Tujia (0.12%) populations (Figure 4B).

Diagnostic stratification identified hematologic disorders as the primary indications for CsA therapy: aplastic anemia (AA) accounted for nearly half of cases (44.73%), followed by acute myeloid leukemia (AML; 12.59%) and acute lymphoblastic leukemia (ALL; 9.8%) (Figure 4C). Non-hematologic applications included liver transplantation (0.53%), systemic lupus erythematosus (SLE; 1.35%), juvenile idiopathic arthritis (JIA; 0.45%), and nephrotic syndrome (0.49%), among others. Notably, this clinical utilization pattern markedly diverged from the meta-analysis findings (Supplementary Table S1), highlighting institution-specific prescribing practices for CsA in pediatric hematology/oncology.

Geospatial analysis demonstrated nationwide patient origins, with disproportionate representation from eastern China’s coastal economic hubs (72.24% collectively): Anhui Province contributed the largest subset (15.91%), followed by Zhejiang Province (14.56%) and Shanghai Metropolis (14.31%) (Figure 4D).

#### 4.2.2 Possible complication profile and infectious burden of pediatric CsA therapy at SCMC

**FIGURE 5:**
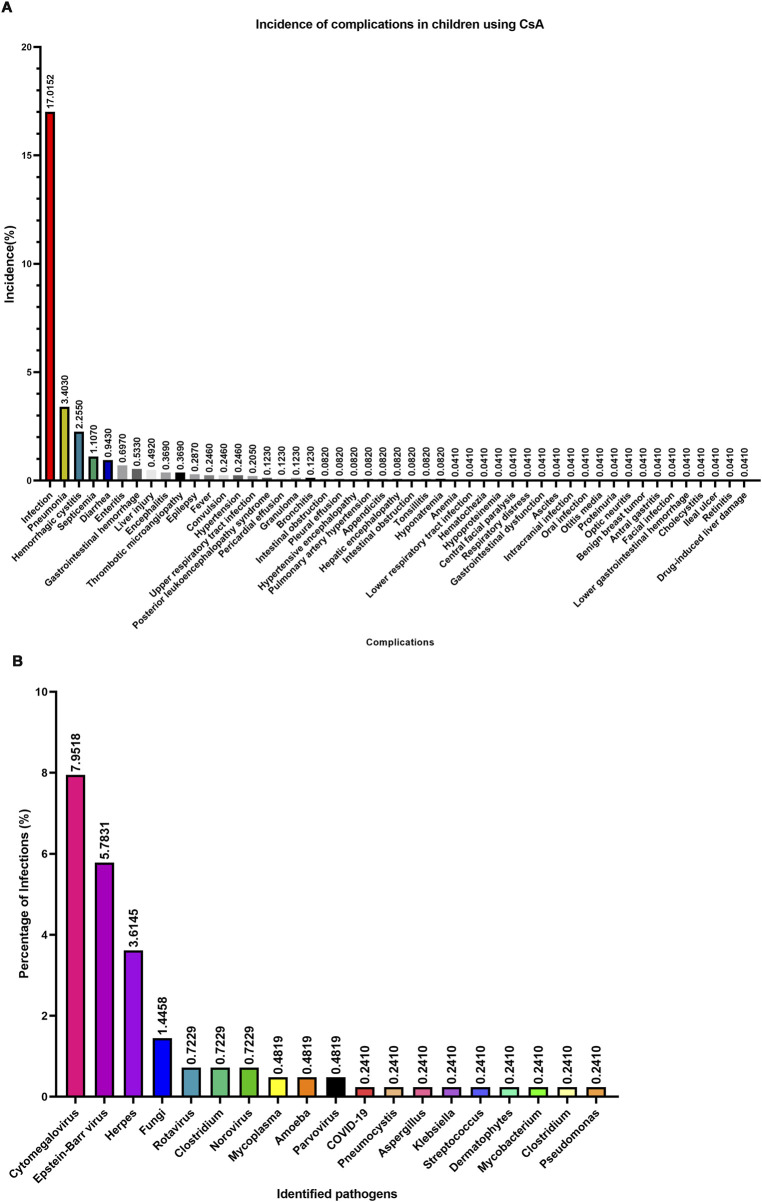
Complications and pathogens associated with CsA treatment in children. **(A)** Complications and their incidence rate. **(B)** Pathogens and their incidence rate.

Systematic analysis revealed 49 distinct complication phenotypes during CsA administration (Figure 5A). The predominant complication spectrum comprised: infectious events (17.02% of total cohort, 55.78% of complication cases), infection-associated sequelae (pneumonia, hemorrhagic cystitis, septicemia, diarrhea, enteritis, and encephalitis), and organ-specific toxicities (liver injury, gastrointestinal hemorrhage, thrombotic microangiopathy, hypertension, and reversible posterior leukoencephalopathy syndrome). Notably, infection-related complications collectively accounted for >95% of all adverse events (Figure 5A), suggesting that infection prevention may be as the paramount safety consideration in pediatric CsA therapy.

Pathogen characterization in 415 infection cases identified 19 microbial agents (Figure 5B): Viral predominance: cytomegalovirus (CMV; 7.95%), Epstein-Barr virus (EBV; 5.78%), Human Herpesvirus (HPV; 3.6%); Bacterial threats: *Clostridium difficile* (recurrent detection)*, Klebsiella pneumoniae,* and *Pseudomonas aeruginosa*; Opportunistic pathogens: Pneumocystis carinii, Aspergillus spp., *Mycoplasma*. These findings suggested that three-tiered infection prevention strategy should prioritize: CMV/EBV prophylaxis (most prevalent viral pathogens), antifungal coverage for *aspergillus* and fungal species, and Gram-negative bacterial surveillance.

#### 4.2.3 Common laboratory test indicators following CsA administration in pediatric patients

**FIGURE 6:**
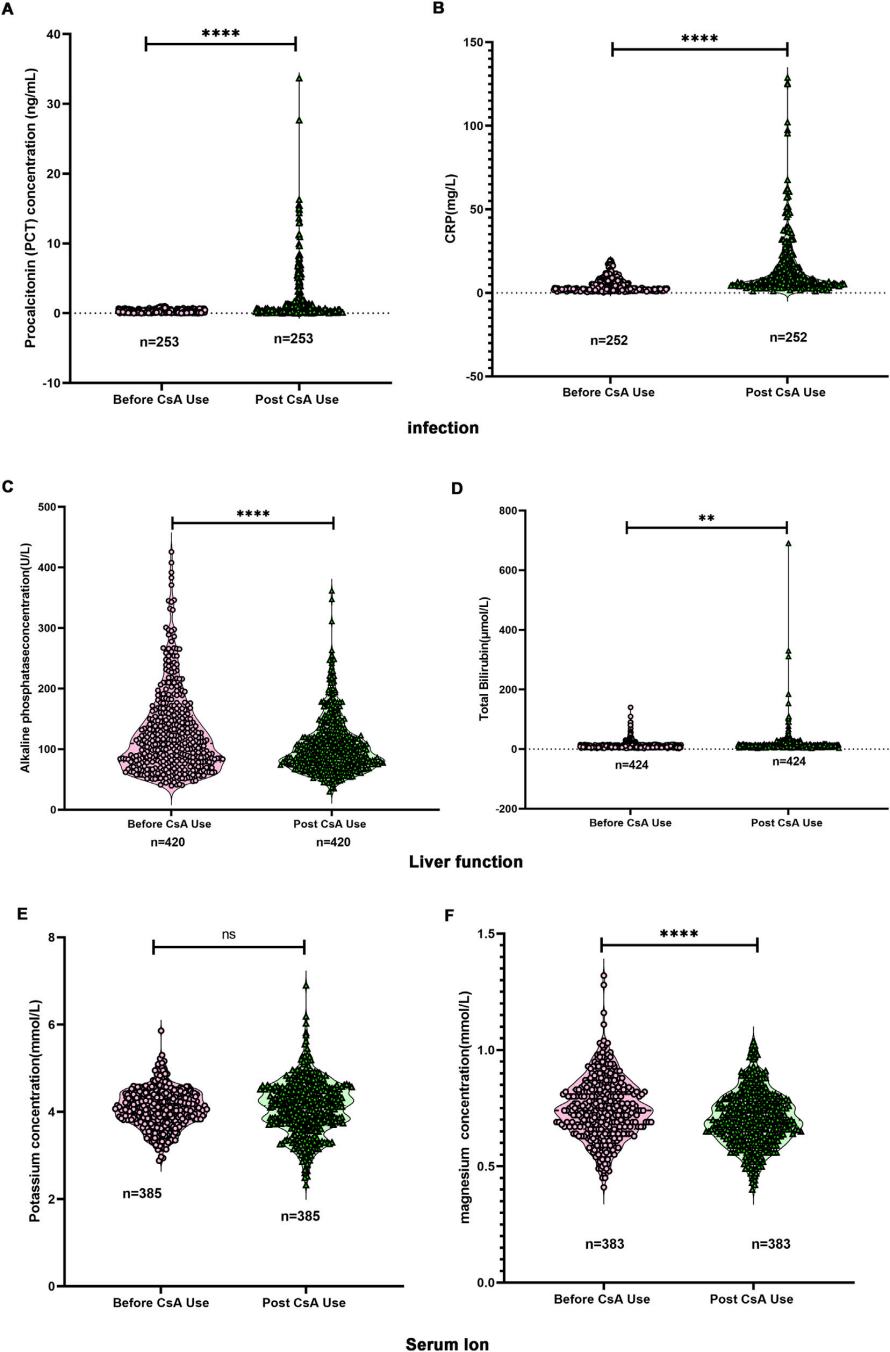
Changes in laboratory testing indicators before and after the use of CsA. **(A)** Changes in procalcitonin (PCT) levels. **(B)** Changes in C-reactive protein (CRP). **(C)** Changes in alkaline phosphatase (ALP). **(D)** Changes in total bilirubin (TB). **(E)** Changes in serum potassium. **(F)** Changes in serum magnesium. Paired Student’s t-test.

To characterize physiological parameter changes during CsA therapy in children, we analyzed laboratory test results from 2,439 pediatric patients. As demonstrated in Figures 6A,B, CsA administration resulted in significant elevations of the infection markers PCT and CRP. Hepatic function parameters showed divergent responses: ALP levels decreased post-treatment (Figure 6C), while TB concentrations increased (Figure 6D). Electrolyte analysis revealed distinct patterns - serum potassium levels remained stable following CsA exposure (Figure 6E), whereas serum magnesium concentrations demonstrated a significant reduction (Figure 6F).

#### 4.2.4 Age-stratified analysis of physiological parameters

Based on established clinical consensus that cardiac maturation reaches completion during adolescence (10–18 years) (Simon et al., 2015; Heidenreich et al., 2022), we stratified our cohort into two age groups: <10 years (pre-adolescent) and ≥10 years (adolescent/young adult) to examine developmental differences in treatment responses.

As shown in Supplementary Figure S4A, CsA administration induced significant PCT elevation in both age groups (p < 0.05). Baseline PCT levels showed no intergroup differences prior to treatment.

CRP responses demonstrated age-dependent baseline variation (Supplementary Figure S4B). While both groups exhibited significant post-CsA CRP elevation (p < 0.001), pretreatment levels were notably higher in the ≥10 years cohort compared to younger patients (6.7 ± 5.8 vs. 3.6 ± 2.6 mg/L, p < 0.001).

Hepatic parameters revealed divergent age-specific responses (Supplementary Figure S4C–D): 1) ALP reduction was exclusively observed in the <10 years group post-treatment, contrasting with stable levels in older patients. Baseline ALP levels were significantly lower in the ≥10 years group; 2) TB increased comparably in both age groups following CsA exposure, with no baseline intergroup difference.

Electrolyte analysis demonstrated differential susceptibility (Supplementary Figure S4E–F): 1) Serum potassium remained stable across age groups post-treatment; 2) Magnesium depletion occurred universally, despite comparable pretreatment levels.

#### 4.2.5 Cardiac functional improvement may associated with CsA therapy in pediatric CHD patients

Among these 2,439 patients, CsA was primarily used for immunosuppression in bone marrow transplantation for leukemia and for autoimmune diseases, (Figure 4C). Sixteen of these patients had CHDs (Supplementary Table S4). The cohort demonstrated the following treatment characteristics: mean body weight 21.4 ± 13.5 kg, CsA dosage 2.41 ± 0.41 mg/kg/time, blood CsA concentration 180.3 ± 65.7 ng/mL, and treatment duration 3.5 ± 3.0 months. Substantial interindividual variability was observed in dosing regimens, with cumulative CsA exposure ranging from 120 mg (2 administrations) to 10,260 mg (91 administrations).

**FIGURE 7:**
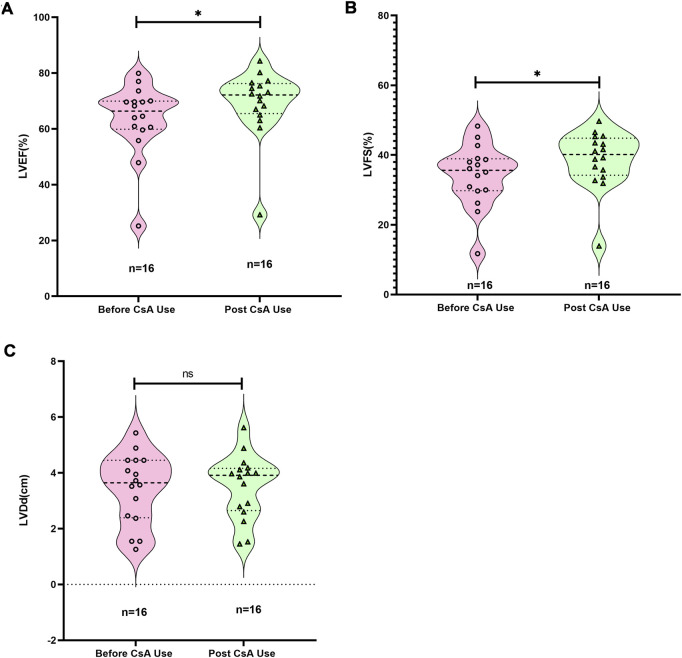
Changes in cardiac function in children before and after the use of CsA. **(A)** Changes in left ventricular ejection fraction (LVEF). **(B)** Changes in left ventricular fractional shortening (LVFS). **(C)** Changes in left ventricular diameter at end-diastolic. Wilcoxon matched-pairs signed rank test.

Notably, 81.3% (13/16) of CHD cases presented with ventricular VO (Supplementary Table S4). Post-treatment echocardiographic analysis revealed significant improvement in systolic function parameters: left ventricular ejection fraction (LVEF) increased by 9.08% ± 4.5% (95% CI: 6.68%–11.48%, p < 0.05) and left ventricular fractional shortening (LVFS) by 13.2% ± 5.4% (95% CI: 10.32%–16.08%, p < 0.05) from Pre-CsA treatment LVEF and LVFS, respectively (Figures 7A,B). However, left ventricular end-diastolic diameter (LVDd) remained stable throughout treatment (Figure 7C). These findings suggest CsA may enhance myocardial contractility in pediatric CHD patients. The decreased myocardial contractility is one of the important characteristics of immature cardiomyocytes (Zhou et al., 2024; Zheng and Ye, 2024).

## 5 Discussion

Contemporary advances in prenatal diagnosis and surgical techniques have enabled over 90% of children with CHDs to survive into adulthood (Van Bulck et al., 2022; Crea, 2023). Nevertheless, this population remains vulnerable to late cardiovascular complications including arrhythmias and heart failure, particularly in complex CHD subtypes such as tetralogy of Fallot and transposition of the great arteries (Cai et al., 2023; O'Leary et al., 2024; Song et al., 2023). The recent development of neonatal CHD animal models has elucidated critical mechanisms by which CHD-specific hemodynamic stressors–RPF and VO–disrupt cardiopulmonary maturation through chronic immune activation (Zhou et al., 2024; Li et al., 2023; Hu et al., 2022; Sun et al., 2021; Cui et al., 2021). Building on these preclinical findings, CsA has shown promise in mitigating hemodynamic stress-induced cardiopulmonary maturation impairment in murine models (Zhou et al., 2024; Li et al., 2023; Hu et al., 2022; Sun et al., 2021; Cui et al., 2021). This therapeutic potential has been formalized in two recent patents (CN 202110673191.8; CN 202211385332.7) detailing CsA-based regimens for CHD-related cardiopulmonary dysfunction. However, the complications of CsA in pediatric use and its effectiveness in treating pediatric CHDs remains unknown.

This study provides a comprehensive analysis of the complications during CsA use in pediatric patients and its potential efficacy in treating CHDs. The research is significant for several reasons: (1) Cardiac Function Improvement: The study is the first to demonstrate that immunosuppressants may be used to ameliorate VO-induced cardiomyocyte maturation deficits, as suggested by significant increases in LVEF and LVFS after CsA treatment. This finding suggests that immunosuppressants might be a potential therapeutic option for pediatric CHD patients, opening a new window for the use of immunosuppressants and providing a novel possible way to reduce late CHD complications. (2) Infection Prevention: The study suggested that infection may be one of the most common complications of CsA use in children, with CMV, EBV, and HPV being the most prevalent pathogens. This suggests the critical need for stringent infection prevention measures, particularly for CMV, during CsA therapy in pediatric populations. (3) Large Sample Size: With 2,439 pediatric patients included, this is one of the largest studies on CsA use in children as far as we know, providing robust data on its safety. The demographic and clinical data offer valuable insights into the patient population, including the predominance of Han ethnicity and the high prevalence of aplastic anemia among the treated children.

Despite its strengths, the study has several limitations: (1)Retrospective Design: The study is retrospective, which inherently limits the ability to establish causality. Prospective studies are needed to confirm the findings and establish a clearer cause-and-effect relationship between CsA use and its outcomes. (2) Limited Sample Size for CHD Patients: Only 16 children with CHDs were included in the study, which is a small sample size. This limits the generalizability of the findings regarding CsA’s efficacy in improving cardiac function in CHD patients. (3) Lack of Control Group: The study lacks a control group, which makes it difficult to compare the outcomes of CsA treatment with other treatments or no treatment. A randomized controlled trial (RCT) would provide more definitive evidence. (4) Short Follow-Up Period: The average treatment duration was 3.5 months, which is relatively short for assessing long-term outcomes and complications. Longer follow-up periods are necessary to evaluate the sustained effects and potential late-onset complications of CsA therapy, including the incidence of arrhythmias. (5) Heterogeneity in CsA Dosage and Administration: There was significant variation in the frequency and cumulative dosage of CsA, which could affect the consistency of the results. Standardized dosing protocols would help in drawing more reliable conclusions. (6) This study did not include pulmonary function data because children with heart diseases do not routinely undergo pulmonary function testing unless specifically indicated. Future prospective studies should incorporate pulmonary function assessments.

Future studies should investigate the long-term outcomes of CsA therapy, including potential late-onset complications and the durability of cardiac function improvement. In addition, comparative studies with other immunosuppressive agents or alternative treatments for CHDs would help determine the relative efficacy and safety of CsA, and Developing and testing standardized protocols for CsA administration in pediatric patients could help minimize variability and improve the consistency of treatment outcomes.

## Data Availability

The original contributions presented in the study are included in the article/Supplementary Material, further inquiries can be directed to the corresponding authors.
