# Supplementary material for: The complications of cyclosporine a in pediatric use and its effectiveness in treating pediatric congenital heart diseases-a meta analysis in combined with a retrospective clinical study
Source: Front Pharmacol. 2025 Nov 27;16:1727970. doi: 10.3389/fphar.2025.1727970 (PMC12695552; doi:10.3389/fphar.2025.1727970)
Supplement: Supplementary file 1 [file Table1.docx]

Table S1 The characteristics of literature included in the meta-analysis

| Author | Publication year | Study disease | Study year | Study districts | N | Age | Gender(Male/Female) |
| --- | --- | --- | --- | --- | --- | --- | --- |
| Alsultan A | 2009 | Severe aplastic anemia | 2003.1-2008.3 | USA | 13 | 9 (3.5–17.5) | NA |
| Goronkova O1 | 2023 | Aplastic anemia | 2016.12-2020.10 | Russia | 49 | 8.7 (2.1-16.8) | 30/19 |
| Goronkova O2 | 2023 | Aplastic anemia | 2016.12-2020.10 | Russia | 49 | 10.5 (2-17.7) | 35/14 |
| Assadi F | 2022 | Steroid resistant nephrotic syndrome | 2019.3-2021.4 | USA | 34 | 3.6 (2.1;5.1) | 14/20 |
| Dorresteijn EM | 2008 | Nephrotic syndrome | 2003.1-2005.6 | Netherlands/Belgium | 12 | 9.2 (3.7–17.5) | 11/1 |
| Iijima K1 | 2013 | Frequently relapsing nephritic syndrome | 2005.4-2009.3 | Japan | 43 | 7.0±4.3 | 32/11 |
| Iijima K2 | 2013 | Frequently relapsing nephritic syndrome | 2005.4-2009.3 | Japan | 42 | 7.1±3.7 | 31/11 |
| Ishikura K1 | 2008 | Frequently relapsing nephritic syndrome | 1996.1-2002.1 | Japan | 24 | Mean age:8.5 | 18/6 |
| Ishikura K2 | 2008 | Frequently relapsing nephritic syndrome | 1996.1-2002.1 | Japan | 20 | Mean age:8.9 | 17/3 |
| Ishikura K | 2010 | Frequently relapsing nephritic syndrome | 2000.1-2005.12 | Japan | 62 | 1.3-15.3 | 48/14 |
| Plank C | 2008 | Steroid resistant nephrotic syndrome | 2001.1-2004.11 | Germany | 15 | 6.22±5.11 | 11/4 |
| Filler G | 2005 | Pediatric renal transplantation | 1996.12-1999.6 | Nine European countries | 93 | NA | NA |
| Hocker B | 2005 | Renal transplantation | NA | Germany | 100 | 8.4±4.9 | 68/32 |
| Offner G1 | 2008 | Pediatric renal transplantation | 2001.5-2006.1 | Germany/France | 100 | 10.7±4.6 | 56/44 |
| Offner G2 | 2008 | Pediatric renal transplantation | 2001.5-2006.1 | Germany/France | 93 | 10.8±4.6 | 62/31 |
| Staskewitz A | 2001 | Renal transplantation | 1996.10- 1999.6 | Germany | 65 | 11.5±3.6 | 42/23 |
| Turconi A | 2005 | Pediatric renal transplantation | NA | Argentina | 18 | 11.9±4.5 | 10/8 |
| Vester U | 1998 | Renal transplantation | 1975-1994 | Germany | 46 | 3.2(0.8-5.9) | 31/15 |
| Harper JH1 | 2000 | Atopic dermatitis | 1995.6 - 1997.12 | UK | 21 | 10.1(3-16) | 19/21 |
| Harper JH2 | 2000 | Atopic dermatitis | 1995.6 - 1997.12 | UK | 19 | 10.0(3-16) |  |
| El-Khalawany MA | 2012 | Severe atopic dermatitis | NA | Egypt | 20 | 10.30±2.82 | 14/6 |
| Ruperto N | 2016 | Juvenile dermatomyositis | 2006.5-2010.11 | 22 countries | 46 | 8.8 (5.0–11.3) | 20/26 |
| Liu AP | 2016 | Chronic immune thrombocytopenia | 1998.1-2015.6 | China | 29 | 5.0(0.5-16.2) | 14/15 |

Goronkova O1:cyclosporin A;Goronkova O2:cyclosporin A+eltrombopag; Goronkova O1 and Goronkova O2 from the same publication

Harper JH1:Short ; Harper JH2: Continuous;Harper JH1 and Harper JH2 from the same publication.

Iijima K1: the cyclosporine target was set to 600–700 ng/ml for the first 6 months and 450–550 ng/ml for the next 18 months; Iijima K2: it was set to 450–550 ng/ml for the first 6 months and 300–400 ng/ml for the next 18 months. Iijima K1 and Iijima K2 from the same publication

Ishikura K1:the dose was adjusted to maintain a slightly lower (60–80 ng/ml) :Ishikura K2: a fixed dose of 2.5 mg/kg/day. Ishikura K1 and Ishikura K2 from the same publication.

Offner G1 and Offner G2 were from the same publication
