# Supplementary figures and images for: The complications of cyclosporine a in pediatric use and its effectiveness in treating pediatric congenital heart diseases-a meta analysis in combined with a retrospective clinical study
Source: Front Pharmacol. 2025 Nov 27;16:1727970. doi: 10.3389/fphar.2025.1727970 (PMC12695552; doi:10.3389/fphar.2025.1727970)

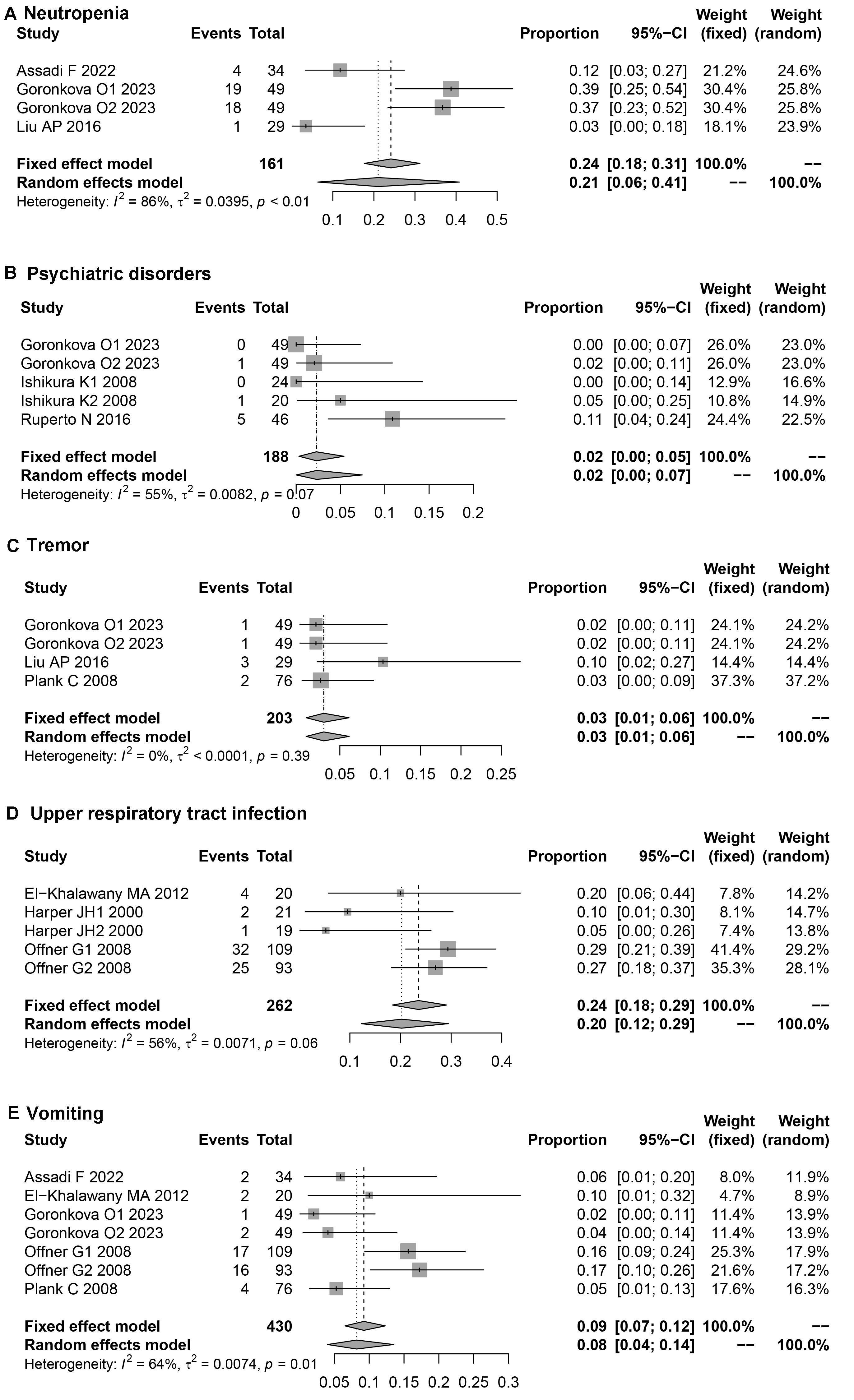

Supplement: Supplementary file 2 [file Image3.tif]

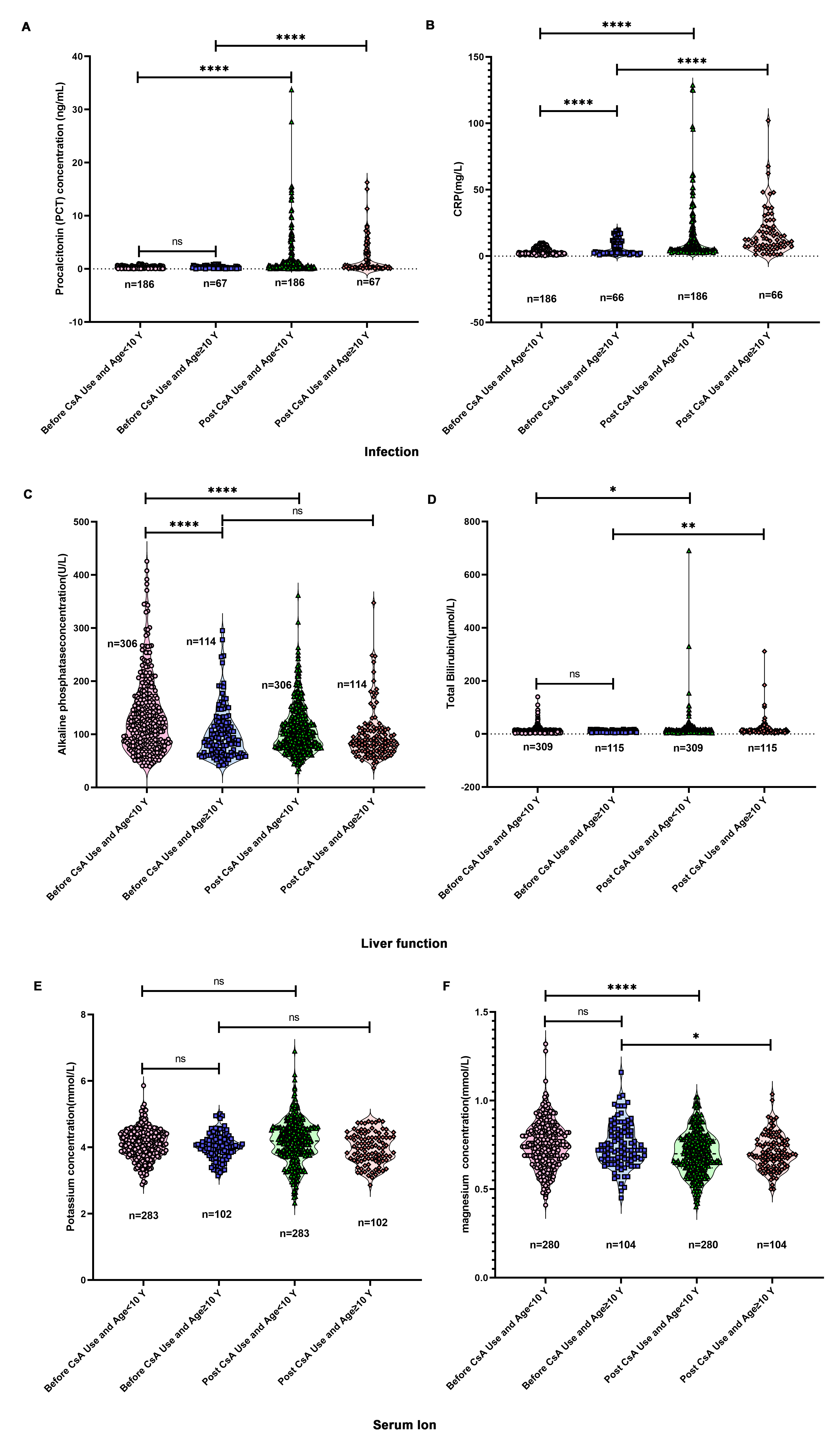

Supplement: Supplementary file 3 [file Image4.tif]

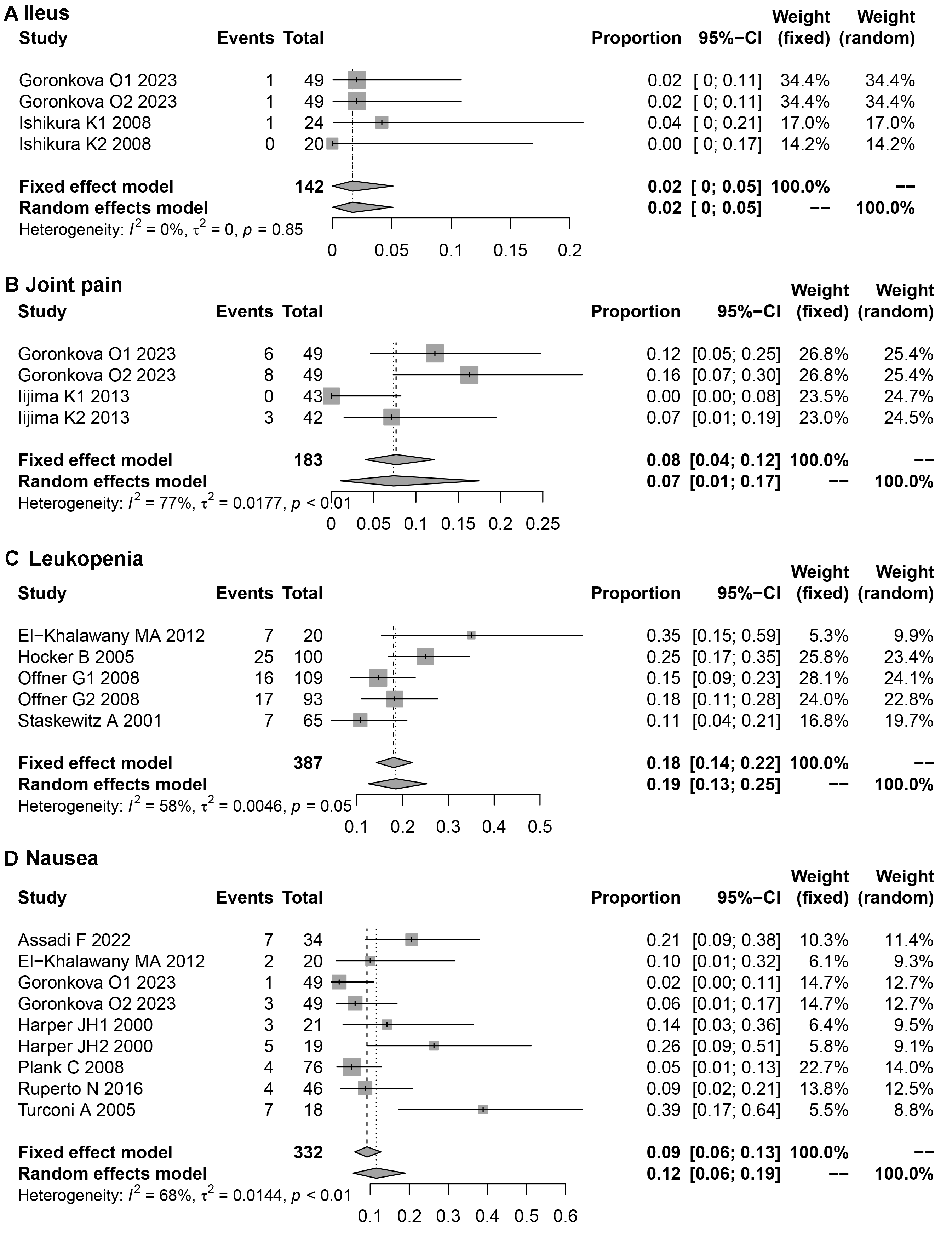

Supplement: Supplementary file 4 [file Image2.tif]

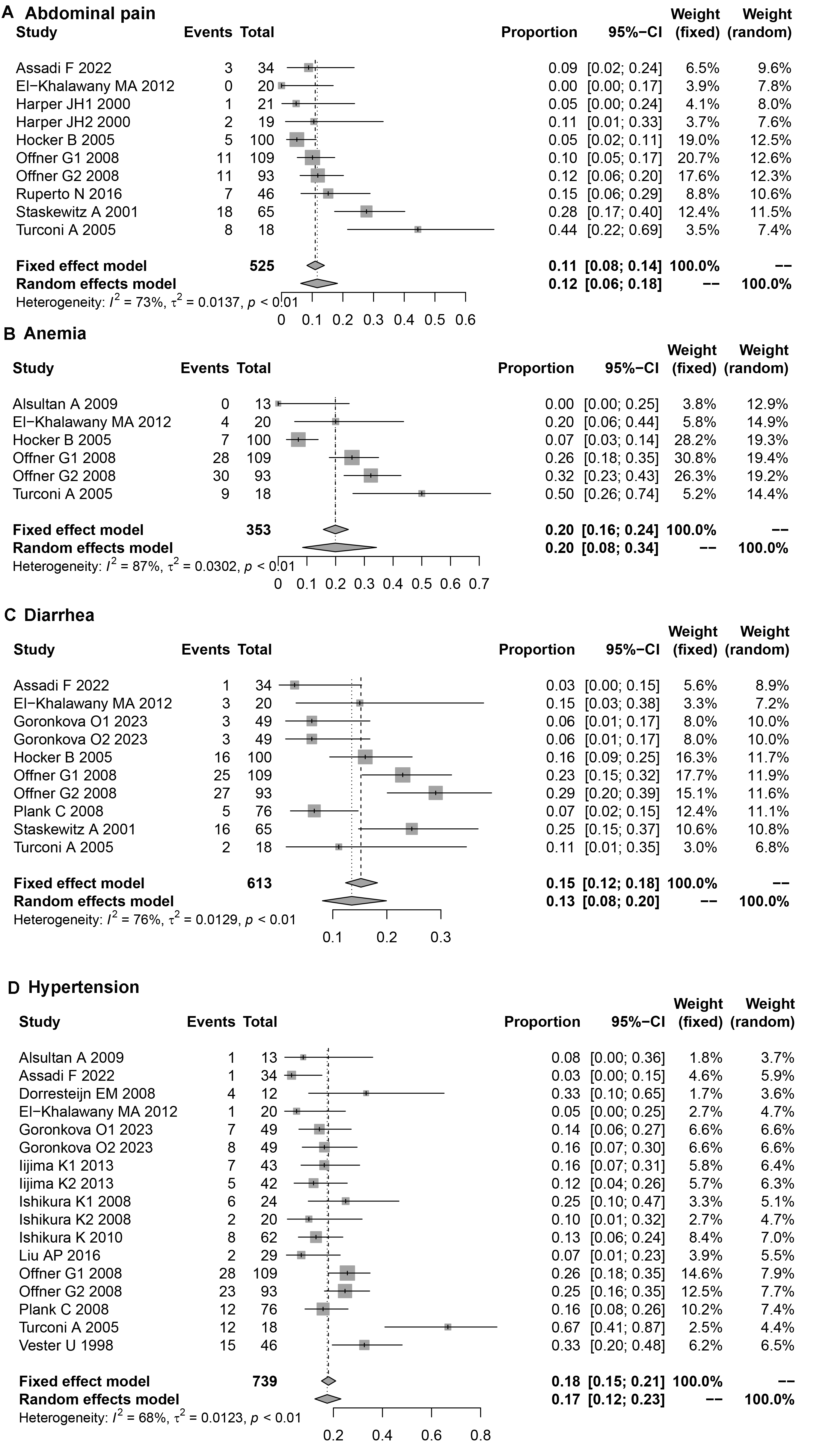

Supplement: Supplementary file 5 [file Image1.tif]
