# Supplementary material for: The complications of cyclosporine a in pediatric use and its effectiveness in treating pediatric congenital heart diseases-a meta analysis in combined with a retrospective clinical study
Source: Front Pharmacol. 2025 Nov 27;16:1727970. doi: 10.3389/fphar.2025.1727970 (PMC12695552; doi:10.3389/fphar.2025.1727970)
Supplement: Supplementary file 6 [file Table2.docx]

Table S2 The combined results of meta-analysis

| Variable | K | N | n | Incidence Rate （P[95%CI]） | Model Type | I^2[95%CI] | Q | P |
| --- | --- | --- | --- | --- | --- | --- | --- | --- |
| Abdominal Pain | 10 | 525 | 66 | 0.1164 [0.0625; 0.1823] | R | 73.4% [49.9%; 85.9%] | 33.82 | <0.0001 |
| Anemia | 6 | 353 | 78 | 0.1990 [0.0850; 0.3422] | R | 86.6% [73.0%; 93.3%] | 37.26 | <0.0001 |
| Diarrhea | 10 | 613 | 101 | 0.1350 [0.0809; 0.1992] | R | 75.6% [54.6%; 86.8%] | 36.82 | <0.0001 |
| Upper Respiratory Tract Infection | 5 | 262 | 64 | 0.2022 [0.1224; 0.2945] | R | 56.4% [0.0%; 83.8%] | 9.17 | 0.0571 |
| Gingival Hyperplasia | 7 | 258 | 36 | 0.1282 [0.0873; 0.1747] | F | 47.4% [0.0%; 77.8%] | 11.41 | 0.0764 |
| Headache | 6 | 150 | 16 | 0.0994 [0.0525; 0.1568] | F | 0.0% [0.0%; 72.0%] | 4.54 | 0.4751 |
| Hirsutism | 11 | 336 | 109 | 0.2834 [0.1838; 0.3940] | R | 76.3% [57.6%; 86.8%] | 42.27 | <0.0001 |
| Hypertension | 17 | 739 | 142 | 0.1747 [0.1245; 0.2306] | R | 67.9% [46.8%; 80.6%] | 49.80 | <0.0001 |
| Ileus | 4 | 142 | 3 | 0.0169 [0.0000; 0.0511 | F | 0.0% [0.0%; 43.9%] | 0.82 | 0.8451 |
| Infection | 9 | 492 | 293 | 0.5576 [0.3117; 0.7900] | R | 96.6% [95.0%; 97.6%] | 232.92 | <0.0001 |
| Joint Pain | 4 | 183 | 17 | 0.0735 [0.0110; 0.1745] | R | 76.5% [35.8%; 91.4%] | 12.79 | 0.0051 |
| Leukopenia | 5 | 387 | 72 | 0.1851 [0.1255; 0.2527] | R | 57.7% [0.0%; 84.3%] | 9.47 | 0.0505 |
| Nausea | 9 | 332 | 36 | 0.1154 [0.0563; 0.1893] | R | 67.5% [34.6%; 83.9%] | 24.65 | 0.0018 |
| Neutropenia | 4 | 161 | 42 | 0.2104 [0.0633; 0.4080] | R | 86.4% [66.8%; 94.4%] | 21.99 | <0.0001 |
| Psychiatric Disorders | 5 | 188 | 7 | 0.0232 [0.0000; 0.0745] | R | 54.7% [0.0%; 83.3%] | 8.83 | 0.0655 |
| Tremor | 4 | 203 | 7 | 0.0301 [0.0083; 0.0613] | F | 0.5% [0.0%; 84.8%] | 3.01 | 0.3894 |
| Vomiting | 7 | 430 | 44 | 0.0820 [0.0400; 0.1352] | R | 63.9% [18.5%; 84.0%] | 16.64 | 0.0107 |

K: Number of included studies; N: Total sample size; n: Number of individuals experiencing the event; Incidence Rate (P[95%CI]): Combined incidence rate and its confidence interval; Model Type: Fixed-effect(F) or random-effect (R) model; I²[95%CI]: Magnitude of heterogeneity and its confidence interval; Q: Q statistic for heterogeneity testing; P: P value for heterogeneity testing.
