# Supplementary material for: The complications of cyclosporine a in pediatric use and its effectiveness in treating pediatric congenital heart diseases-a meta analysis in combined with a retrospective clinical study
Source: Front Pharmacol. 2025 Nov 27;16:1727970. doi: 10.3389/fphar.2025.1727970 (PMC12695552; doi:10.3389/fphar.2025.1727970)
Supplement: Supplementary file 7 [file Table3.docx]

Table S3 Publication bias

| Variable | Egger’s test | | Begg's test | |
| --- | --- | --- | --- | --- |
|  | t | P | Z | P |
| Abdominal Pain | 0.4399 | 0.6716 | 0.4472 | 0.6547 |
| Anemia | 0.0028 | 0.9979 | -0.1879 | 0.8510 |
| Diarrhea | -1.4351 | 0.1892 | -1.2622 | 0.2069 |
| Upper Respiratory Tract Infection | -3.5663 | 0.0377 | -1.9596 | 0.0500 |
| Gingival Hyperplasia | 2.0833 | 0.0917 | 1.9524 | 0.0509 |
| Headache | 0.1950 | 0.8549 | 0.7651 | 0.4442 |
| Hirsutism | -1.6438 | 0.1346 | -1.3234 | 0.1857 |
| Hypertension | -0.3580 | 0.7253 | -0.4952 | 0.6205 |
| Ileus | -0.0334 | 0.9764 | -0.3826 | 0.7021 |
| Infection | -1.2053 | 0.2673 | -0.7338 | 0.4631 |
| Joint Pain | -1.5996 | 0.2508 | -1.0835 | 0.2786 |
| Leukopenia | 0.7733 | 0.4957 | 0.0000 | 1.0000 |
| Nausea | 3.4782 | 0.0103 | 2.8304 | 0.0046 |
| Neutropenia | -32.1660 | 0.0010 | -1.8058 | 0.0710 |
| Psychiatric Disorders | 0.0785 | 0.9424 | 0.7579 | 0.4485 |
| Tremor | 1.8334 | 0.2082 | 0.3826 | 0.7021 |
| Vomiting | -1.3089 | 0.2475 | -0.3038 | 0.7613 |
