# Supplementary material for: The complications of cyclosporine a in pediatric use and its effectiveness in treating pediatric congenital heart diseases-a meta analysis in combined with a retrospective clinical study
Source: Front Pharmacol. 2025 Nov 27;16:1727970. doi: 10.3389/fphar.2025.1727970 (PMC12695552; doi:10.3389/fphar.2025.1727970)
Supplement: Supplementary file 8 [file Table4.docx]

| Patient NO | Body weight (kg) | Number of administrations | Cumulative dose (mg) | Average dose per administration (mg) | Average dose per kilogram (mg/kg) | CsA Blood concentration(ng/ml) | Diagnosis | With VO | Duration of treatment (months) |
| --- | --- | --- | --- | --- | --- | --- | --- | --- | --- |
| 1 | 16 | 6 | 300 | 50.00 | 3.13 | 251.6 | AA with PH | Unclear | 1 |
| 2 | 12.5 | 32 | 1200 | 37.50 | 3.00 | 141.8 | AA with Cardiac Dysfunction | YES | 4 |
| 3 | 30.0 | 75 | 5250 | 70.00 | 2.33 | 144.5 | JRA with Pericarditis | Unclear | 7 |
| 4 | 15 | 35 | 875 | 25.00 | 1.67 | 178.1 | AA with MR | YES | 3 |
| 5 | 10.5 | 30 | 700 | 23.33 | 2.22 | 231.3 | Kawasaki Diseases with ASD and PFO | YES | 4 |
| 6 | 52 | 91 | 10260 | 112.75 | 2.17 | 111.2 | SLE with AVI | YES | 12 |
| 7 | 37.9 | 4 | 300 | 75.00 | 1.98 | 111.7 | ALL with AVI | YES | 0.5 |
| 8 | 25 | 74 | 4525 | 61.15 | 2.45 | 228.1 | AA with AR | YES | 7 |
| 9 | 42.3 | 2 | 200 | 100.00 | 2.36 | 189.6 | HLH with PFO and PH | YES | 0.5 |
| 10 | 4.5 | 12 | 120 | 10.00 | 2.22 | 126.0 | AA with VSD, PFO, PDA, and PH | YES | 1.5 |
| 11 | 23 | 13 | 925 | 71.15 | 3.09 | 189.3 | AA with PH | Unclear | 1.5 |
| 12 | 3.8 | 30 | 300 | 10.00 | 2.63 | 212.9 | SLE with IAA/VSD/PFO/PDA/PH | YES | 4 |
| 13 | 20.0 | 8 | 400 | 50.00 | 2.50 | 132.2 | JRA with TOF/ASD/LSVC-CS | YES | 4 |
| 14 | 21.0 | 8 | 320 | 40.00 | 1.90 | 142.8 | SLE with TOF/ASD | YES | 2 |
| 15 | 17.0 | 12 | 480 | 40.00 | 2.35 | 360.3 | AA with D-TGA/VSD/PS | YES | 2 |
| 16 | 11.5 | 16 | 480 | 30.00 | 2.61 | 133.5 | AA with MR | YES | 2 |
| Mean±SD | 21.4±13.5 | 28±28.03 | 1664.7±2751.5 | 50.4±29.8 | 2.41±0.41 | 180.3±65.7 | / | / | 3.5±3.0 |
| Note: AA:Aplastic Anemia;PH:Pulmonary Hypertension;JRA:Juvenile Rheumatoid Arthritis;MR:Mitral Regurgitation;ASD:Atrial Septal Defect;PFO:Patent Foramen Ovale;  SLE:Systemic Lupus Erythematosus;ALL:Acute Lymphoblastic Leukemia;AVI:Aortic Valve Insufficiency; HLH:Hemophagocytic Lymphohistiocytosis;PDA:Patent Ductus Arteriosus;IAA:Interrupted Aortic Arch;TOF:Tetralogy of Fallot;LSVC-CS:Left Superior Vena Cava connecting to Coronary Sinus;D-TGA:Dextro-Transposition of the Great Arteries; VSD:Ventricular Septal Defect | | | | | | | | | |

Table S4 Utilization of CsA in Pediatric Patients with Cardiac Diseases
